# Effects of modified aerobic training on muscle metabolism in individuals with peripheral arterial disease: a randomized clinical trial

**DOI:** 10.1038/s41598-019-52428-7

**Published:** 2019-11-04

**Authors:** Débora Pantuso Monteiro, Giane Amorim Ribeiro-Samora, Raquel Rodrigues Britto, Danielle Aparecida Gomes Pereira

**Affiliations:** 1 Graduate Program in Rehabilitation Sciences, Universidade Federal de Minas Gerais, Avenida Presidente Antônio Carlos, 6627 - Pampulha, Belo Horizonte, MG CEP 31270-901 Brazil; 2 Department of Physical Therapy, Universidade Federal de Minas Gerais, Avenida Presidente Antônio Carlos, 6627 - Pampulha, Belo Horizonte, MG CEP 31270-901 Brazil

**Keywords:** Cardiology, Health care

## Abstract

The primary objective of this study was to compare the effects on muscle metabolism of two types of aerobic training, with and without a load on the lower limbs, in adults with peripheral arterial disease (PAD). A simple blind randomized clinical trial was conducted using two groups: conventional aerobic (CG) and modified aerobic with a load on the lower limbs (MG). Both groups underwent training by walking three times a week over a 12-week period. The ratings of muscle metabolism were determined after a treadmill test with constant velocity and inclination concomitant with the use of near infrared spectroscopy (NIRS). Altogether 40 individuals with PAD (CG = 65.45 ± 10.60 and MG = 63.10 ± 10.54) were included in the study. After the intervention, in both groups, there was a reduction in the relative time to recovery (p = 0.002), an improvement in the re-oxygenation rate (p = 0.017), an increased time of resistance after reaching the lowest muscle oxygen saturation (StO_2_) (p < 0.001), an increase in the distance walked (p < 0.001), and an improvement of the walking economy relative to StO_2_ (p < 0.001). After 12 weeks of training, an improvement in the deoxygenation rate was observed in both groups (p = 0.002), but with a greater magnitude in the CG (p = 0.017). Only the CG presented an increase in time to reach the lowest StO_2_ on the treadmill after the intervention (p = 0.010). The traditional aerobic training was superior to the modified training in relation to the improvement of muscle metabolism in patients with PAD.

## Introduction

Individuals with peripheral arterial disease (PAD) have lower limb muscle ischemia due to imbalances between perfusion and metabolic demand^1,2^, with a direct impact on the ability to walk resulting in significant functional limitations^3^. The pathophysiological process of PAD is multifactorial and includes, in addition to arterial obstruction, endothelial dysfunction with poor blood flow distribution, mitochondrial dysfunction associated with worsening peripheral oxygen utilization capacity and increased inflammatory activity^4^.

Due to the musculoskeletal changes of PAD, such as loss of muscle mass and consequent reduction of the capacity to produce strength and resistance^5–8^, resistance muscle training has been suggested as a possible intervention in the treatment of individuals with the disease^6,9^. There is no consensus in the literature about the effects of this type of training in patients with PAD due to the heterogeneity of protocols used^5,6,9–12^. Therefore, to date, it is suggested that resistance training be a complementary intervention to traditional aerobic training. Physical exercise using walking, along with the use of shin weights in the lower limbs, may be an alternative way of imposing overload functionally, considering that individuals with PAD present with a significant limitation in performance during submaximal activities such as walking^4^. Although this training is a viable therapeutic option in the treatment of individuals with PAD, the effects of this type of program on muscle metabolism have not been described in the literature.

Using near-infrared light spectroscopy (NIRS) it is possible to verify, in real time, muscle ischemia triggered by ambulation or exercise by adjusting the oxyhemoglobin (HbO_2_), deoxyhemoglobin (HHb) and tissue saturation (StO_2_) variables^13,14^. The absolute values of oxygen saturation (StO_2_) at rest are similar between individuals with PAD and healthy individuals^15^. However, during physically active individuals with PAD reach significantly lower values of StO_2_, present a sudden fall in StO_2_ at the beginning of the exercise, and present a longer recovery time compared to healthy individuals^13–16^. The recovery time of StO_2_ in diabetic individuals with PAD that are unable to complete a five-minute treadmill test is significantly longer than the recovery time of individuals that are able to complete the same test^15,17^. The evaluation of individuals with PAD with the aid of NIRS makes it possible to simultaneously analyze the effects of limiting or compensatory factors on perfusion, muscle metabolism and functional capacity^18^. Given the alterations secondary to PAD, it is important to increase our knowledge of the chronic effects of the different types of training (aerobic and resisted muscle) on the muscular metabolic response.

Therefore, the primary objective of this study was to evaluate the effects of a modified training program on muscle metabolism, using walking concomitant with the use of lower limb overload in individuals with peripheral arterial disease, and compare it to traditional aerobic walking with respect to limiting symptoms of ischemia. The secondary objective was to compare the functional capacity response to the two types of training. The hypothesis of the present study was that patients with PAD who performed the modified aerobic with a load on the lower limbs would present greater gains in functional capacity, metabolism and muscular performance than subjects submitted to traditional aerobic walking training.

## Materials and Methods

### Study design

This study was approved by the Research Ethics Committee of Universidade Federal de Minas Gerais on February 15, 2016 (CAAE registration 51274515.4.0000.5149) and was registered at http://www.isrctn.com (ISRCTN 44928994). The project summary and consort check list are shown in supplementary information. All methods were performed in accordance with the relevant guidelines and regulations. All participants were duly informed about the study, including possible risks and benefits of the interventions, before signing the informed consent form. The present study is a randomized, single-blind clinical trial. It was not possible to blind the participants and the professionals responsible for the intervention due to the obvious difference between the two types of intervention. Therefore, only the evaluators were blinded and did not know the allocation of the individuals to the intervention groups. Participants were included in the study upon meeting the inclusion criteria. After selection and evaluation, the project coordinator referred the subjects to the professional responsible for the intervention, who, from a, block-generated sequential list, randomly allocated participants to one of two groups, the conventional aerobic group (CG) or the modified aerobic group concomitant with the use of lower limb (MG) overload. The project coordinator was responsible for generating the random allocation sequence, performed in blocks of four at www.randomization.com.

### Sample

From February 2016 to March 2017 adults with PAD and intermittent claudication were enrolled in the study, from the Cardiology and Vascular Surgery Clinic, Clinical Hospital, Universidade Federal de Minas Gerais. Individuals with PAD were included in the study, regardless of sex, according to the following inclusion criteria: (1) presenting an ankle-brachial index (ABI) at rest of less than 0.9 and (2) no pain at rest. The exclusion criteria of the study were: (1) participation in a supervised exercise program in the last six months or (2) presence of diseases or complications that impeded training such as heart failure, unstable angina, arrhythmia, decompensated diabetes (capillary glycemia greater than 250 mg/dl), or signs of hemodynamic instability.

### Measures

**Figure 1:**
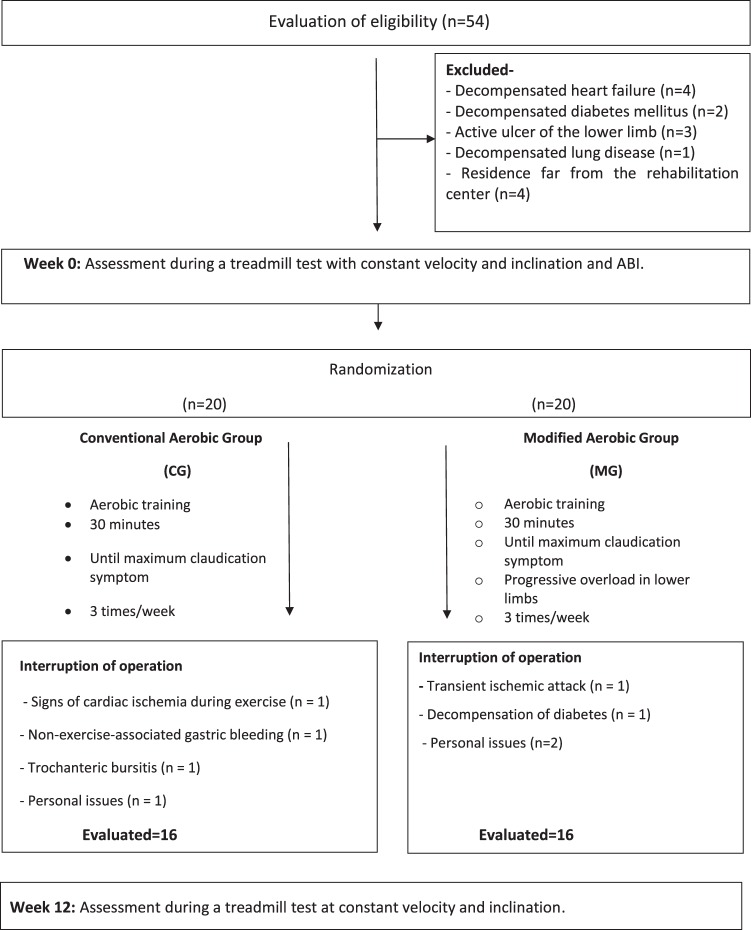
Flowchart of the study - numbers of participants who were randomly assigned, received intended treatment, and were analysed for the primary outcome.

Two evaluations were performed, the first was prior to the intervention, and the second was at 12 weeks after supervised training (Fig. 1). In the evaluation, in order to characterize the sample, clinical data were collected by interview regarding the presence of diabetes mellitus, beta-blocker use, use of cilostazol, smoking, level of obstruction, presence of clinical signs of chronic venous insufficiency, body mass index, and resting ABI. The body mass index was calculated from the weight and height of the individuals measured using an anthropometric scale (Filizola Indústria Ltda, São Paulo-SP, Brazil). A handheld Doppler instrument (DV-2001, MEDPEJ®) were used to measure resting ABI on the left and right sides, obtained using the highest ankle systolic blood pressure (SBP) divided by the highest brachial SBP. The participant remained in the supine position for a period of 15 minutes before the measures begin. A specific upper limb cuff was used to assess the systolic pressures of both limbs. In both lower limbs, SBP was measured from the posterior tibial and dorsal arteries of the foot^19^. In addition, the treadmill test was performed at a constant velocity and slope (3.2 km/h and 10% inclination)^20^. NIRS (Artinis®, Portamon system, The Netherlands) was used to evaluate the changes of StO_2_ and HHb of the medial gastrocnemius muscle during the arterial occlusion maneuver, and during the treadmill test using Oxysoft software (Artinis®). After the ABI measurement, the NIRS sensors were positioned in the medial region of the gastrocnemius muscle at the level of the largest circumference, and fixed with plastic film and an elastic band^18,21^. The data were initially obtained at a frequency of 10 Hz. With the individual positioned in the dorsal decubitus position after initial stabilization of the measurement, the baseline value of StO_2_ was recorded, and the arterial occlusion maneuver was initiated. This maneuver was performed with a cuff positioned between the medial and distal third of the individual’s thigh. The cuff was inflated above 250 mmHg, but to a maximum of 280 mmHg^22–24^, and maintained for a period of five minutes, until the measures stabilized. This procedure worked as a physiological calibration, creating a functional scale that enabled a better comparison between different individuals, as the variables HbO_2_ and HHb were provided by the software in arbitrary units^22,25^. The NIRS device was maintained in the lower limb of the patient until recovery after the treadmill test.

To perform the treadmill test, the participant was instructed to walk for as long as possible until experiencing the moderate-to-maximum claudication symptoms. One minute of warm-up was carried out on the treadmill, in which there was a progressive increase of speed and inclination until 3.2 km/h speed and 10% incline was reached. One to two minutes of active recovery was maintained, in accordance with the tolerance of the individual, with a speed of 2.0 km/h and 0% inclination, from the moment in which the individual experienced the moderate-to-maximum claudication symptoms.

### Study variables

**Figure 2:**
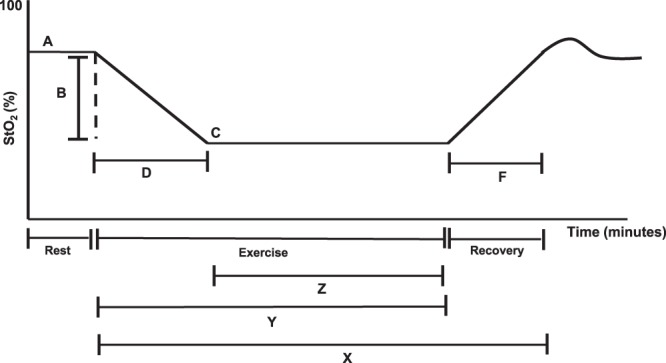
Variables obtained by NIRS during the treadmill test for peripheral arterial disease. Adapted from Boezeman *et al*., 2016. A = StO_2_ value at rest; B = StO_2_ delta: StO_2_ variation during exercise; C = lower saturation value reached during exercise; D = time to reach the lowest saturation; Z = time to resistance after reaching lower StO_2_; X = time taken to recover baseline StO_2_ from the beginning of the test; Y = test duration time; X/Y = Relative recovery time of StO_2_ on the treadmill/; B/D = deoxygenation rate; B/X = re-oxygenation rate; B/(X/Y) = relative re-oxygenation rate during the treadmill test; distance traveled on the treadmill/B = walking economy relative to StO_2_.

The maximum distance traveled in the treadmill test at a constant velocity and inclination, and the variables obtained from the NIRS assessment during the arterial occlusion maneuver and the treadmill test were established as study variables. The variables obtained during the arterial occlusion maneuver were: recovery time of StO_2_ after occlusion, delta/variation of HHb during occlusion and delta/variation of StO_2_ during occlusion. The variables obtained from the treadmill test were: delta/variation of HHb, delta/variation of StO_2_, time to reach lower StO_2_, time to resistance after reaching lower StO_2_, relative time of recovery on the treadmill, deoxygenation rate, re-oxygenation rate, relative re-oxygenation rate, walking economy relative to HHb (meters/delta HHb), and walking economy related to StO_2_. The variables related to StO_2_ are described in Fig. 2.

### Training protocols

The aerobic exercise training was performed three times per week, over a 12-week period by volunteers from both groups. The CG performed traditional training, while the MG performed training with progressive overload in the lower limbs. In both groups, the walking exercise was performed at an acceptable intensity until the moderate-to-maximum claudication symptoms occurred, allowing periods of recovery. As soon as there was a resolution of the claudication symptoms, a new walk was begun. The time required for the resolution of symptoms between each walk was disregarded in recording the total activity time, which should have been 30 minutes. Warm-ups and recovery were performed at the beginning and end of each walk, respectively, by both groups. The following parameters in all sessions were recorded: time to symptom onset, time to the onset of limiting pain, rest time required for symptoms to disappear, and total distance walked by the volunteer in each session. The training supervision was made by physical therapists. They were responsible for monitoring the hemodynamic response, progression of the protocol, as well as providing standardized instructions for the patient to reach moderate-to-maximum claudication symptoms during training.

### Conventional aerobic training

The aerobic training of the CG was started with walking on the floor for 30 minutes. From the moment that the individual ceased to report a moderate-to-maximum claudication symptoms within 30 minutes, treadmill training on the treadmill without inclination, was started at the average velocity achieved during the last walking session on the floor. A progressive rise of 0.2 km/h in velocity was performed from the moment the individual ceased to report moderate-to-maximum claudication symptoms within 30 minutes on the treadmill.

### Modified aerobic training

The aerobic walking training performed by the MG included a progressive overload on the lower limbs through the addition of ankle weights. Training was started on the floor, at first without a load on lower limbs. The loads were added gradually, according to the load progression protocol. From the time the individual reached the minimum time of 15 minutes of walking on the floor without experiencing a moderate-to-maximum claudication symptoms, ankle weights were added, progressively, adding 0.5 kilo in each lower limb up to 2 kilos and taking into account the time and the absence of a moderate-to-maximum claudication symptoms. From the moment that the individual reached the minimum time of 15 minutes with an overload of 2 kilos while walking on the ground without reach moderate-to-maximum claudication symptoms, training was started on the treadmill. The treadmill training started with the average speed reached on the floor, but without overload on the lower limbs. Weights were added at the ankles to increase the overload, progressively, evolving from 0.5 kg in each lower limb up to 2 kg, in the same way as in the floor training. A progressive rise of 0.2 km/h in the speed of the treadmill was followed, if the individual did not reach moderate-to-maximum claudication symptoms within 15 minutes after the addition of the load of 2 kilos.

### Statistical analysis

The Anderson-Darling test was used to evaluate the normal distribution of the continuous variables. The descriptive analysis of the data and the results are expressed as mean ± standard deviation, or as absolute and relative frequencies.

The comparisons of the categorical variables, to characterize the groups, were performed using Fisher’s exact test (2 × 2 tables) or the Cramer’s V coefficient (asymmetric tables). To evaluate the differences between the groups (conventional and modified), the situation (pre- and post-intervention treadmill tests), and the interaction between the groups and the situation, the Linear Mixed Model was used. The dependent variables “groups” and “situation” were entered into the model as fixed effects, and the “subjects” was entered as a random effect. For the choice of the best model, the values for the maximum restricted likelihood (−2 restricted log likelihood) were used, the diagonal covariance structure was used for the repeated measures, and the first order autoregressive structure was used for random effects. Compared to the intention-to-treat analysis, this method provides better estimates for lost data and addresses individual differences more adequately^26,27^. An alpha value of 5% was set for statistical significance. The data were analyzed using the statistical software Statistical Package for the Social Sciences - (SPSS, Inc., USA, version 15.0).

### Sample size calculation

Initially, the sample size was calculated considering the variable total distance on the treadmill test as the most important variable for the analysis^28^. From previous studies of individuals with PAD with similar characteristics to the subjects allocated for this study^29^, the sample size calculated was 70 participants per group, setting a power of 80% and an alpha error of 5%. In a second moment, from a pilot study with 12 individuals in each group, the sample size was recalculated to represent the smallest size of effect for the estimation of the n sample size. To perform the calculation, an alpha error of 5% and a power of 0.80 were set, and the size of the effect of the ANOVA (f) was estimated by the equation: $(f=\sqrt{\frac{S{Q}_{A}}{S{Q}_{e}}})$, where SQ_A_ =  the sum of the squares of the respective source of variation and SQ_E_ = the sum of the squares of the errors^30,31^. From the sample size calculation, 18 individuals were required per group. Considering the potential subject losses, 10% was added to the n calculated, totaling 20 individuals per group.

## Results

**Figure 3:**
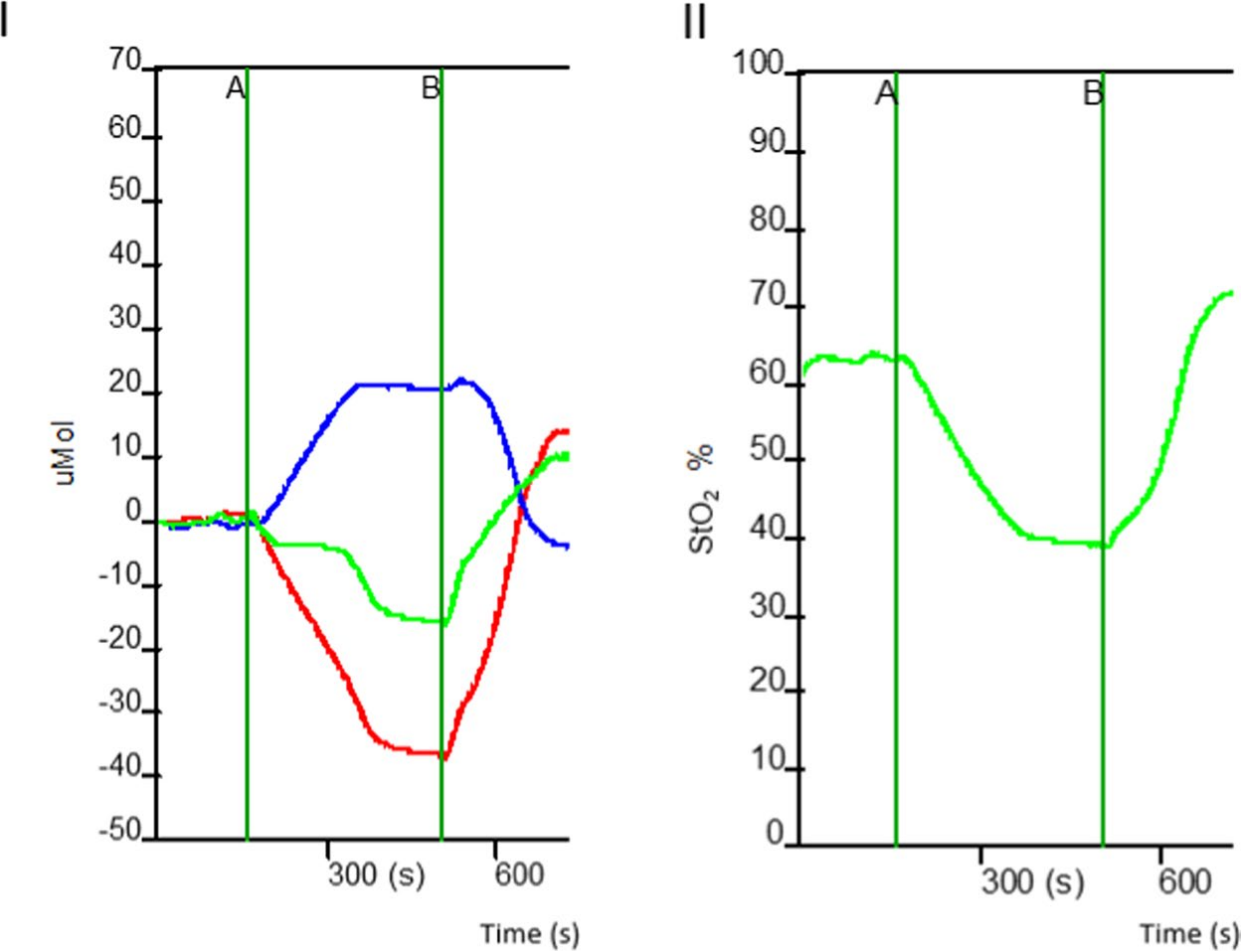
Data from one participant exemplifying the mean changes of the NIRS variables during (A to B) and after (after B) the arterial occlusion maneuver. In the left pannel (I) the red line represents oxyhemoglobin (HbO2); the blue line represents deoxyhemoglobin (HHb) and the green line represents the total hemoglobin (tHb). In the right pannel (II) green line represents tissue saturation (StO2).

Table 1 presents the clinical characteristics of the population sample included in the study. Initially, 54 volunteers with PAD and claudication, were eligible for the study. After applying the inclusion and exclusion criteria, 40 individuals were included. Over the course of the study, eight total participants abandoned treatment, comprising four from each group. The flowchart of the study is shown in Fig. 1. Figure 3 shows the data from one participant exemplifying the mean changes of the NIRS variables during and after the arterial occlusion maneuver.

**Table 1:** Characterization of the sample (n = 40).

		Conventional group (n = 20)	Modified Group (n = 20)	p value
Sex, n (%)	Men	14 (70%)	14 (70%)	0.999
	Women	6 (30%)	6 (30%)	
Age (years)		65.45 ± 10.60	63.10 ± 10.54	0.486
BMI (kg/m^2^)		28.54 ± 4.60*	25.78 ± 4.38	0.038
ABI	Right	0.62 ± 0.17	0.61 ± 0.18	0.531
	Left	0.61 ± 0.17	0.62 ± 0.18	0.689
Smoker, n (%)	Yes	4 (20%)	7 (35%)	0.592
	No	4 (20%)	4 (20%)	
	Ex-smoker	12 (60%)	9 (45%)	
Diabetes Mellitus, n (%)	Yes	9 (45%)	6 (30%)	0.514
Use of beta-blockers, n (%)	Yes	11 (55%)	8 (40%)	0.527
Use of Cilostazol, n (%)	Yes	3 (15%)	10 (50%)*	0.041
Level of Obstruction, n (%)	Superficial Femoral Aortoiliac Unspecified	10 (50%)	11 (55%)	0.289
		4 (20%)	7 (35%)	
		6 (30%)	2 (10%)	
Chronic venous insufficiency (CVI) - CEAP classification, n (%)	Absence Mild CVI (Classes 1, 2 and 3) Moderate to severe CVI (Classes 4, 5 and 6)	1 (5.9%)	2 (10.5%)	0.886
		5 (29.4%)	7 (36.8%)	
		11 (64.7%)	10 (52.6%)	

Data are presented as mean ± standard deviation, or as absolute and relative frequency (%). BMI: Body Mass Index; ABI: ankle-brachial index; CEAP: Clinical Etiology Anatomy Pathophysiology Classification of Chronic Venous Disease; *p < 0.05.

No difference in exercise intensity, characterized by the mean percentage of predicted maximum heart rate for age that was reached by both groups during the sessions (73.77 ± 12.1% in the conventional group and 72.42 ± 10.52% in the modified group, p = 0.740), was observed in the present study. Adherence to treatment was not different between the two groups (91.17 ± 6.39% in the conventional group and 89.05 ± 10.85% in the modified group; p = 0.894). There was no difference in ABI between the groups, or between the pre- and post-intervention situations (CG pre-intervention: right ABI 0.63 ± 0.17 and left ABI 0.63 ± 0.17; CG post-intervention: right ABI 0.65 ± 0.2 and left ABI 0.68 ± 0.16; GM pre-intervention: right ABI 0.62 ± 0.18 and left ABI 0.63 ± 0.18, and post-intervention: right ABI 0.63 ± 0.16 and left ABI 0.62 ± 0.15; right ABI effect group p = 0.531 and effect situation p = 0.070; left ABI effect group p = 0.689 and effect situation p = 0.101).

The mean changes of the NIRS variables during the occlusion maneuver and during the pre- and post-intervention treadmill tests are described in Tables 2 and 3, respectively. In Table 3, one can observe significant differences between the groups in the training response time to reach the lower StO_2_ on the treadmill, and in the deoxygenation rate. An improvement in the functional capacity of the individuals of both groups was observed on the treadmill test after the intervention (CG pre-intervention 183.98 meters 95% CI 115.44–293.19, and CG post-intervention 1669.64 meters 95% CI 1210.41–2303.09; GM pre-intervention 173.95 meters 95% CI 111.78–270.68, and GM post-intervention 1154.48 meters 95% CI 690.97–1928.84; effect group p = 0.385, effect situation p < 0.001, and effect interaction p = 0.444).

**Table 2:** Comparison of mean changes of the NIRS variables between the groups concerning the occlusion maneuver, before and after the intervention.

Variables	Conventional Group		Modified Group		Effect group (p-value)	Effect situation (p-value)	Effect interaction (p-value)
	Pre (n = 20)	Post (n = 16)	Pre (n = 20)	Post (n = 16)			
Recovery Time of occlusion (s)	106.11 (67.23 to 144.99)	179.33 (−138.83 to 97.49)	135.00 (98.12 to 171.88)	381.25 (73.19 to 689.31)	0.299	0.153	0.434
Delta HHb of occlusion (a.u.)	14.74 (11.21 to 18.26)	11.03 (8.20 to 13.87)	13.99 (10.55 to 17.43)	14.34 (11.53 to 17.14)	0.546	0.157	0.089
Delta StO_2_ of occlusion (%)	−21.07 (−23.93 to −8.21)	−19.65 (−23.11 to −16.19)	−21.89 (−24.68 to −9.10)	−23.53 (−26.89 to −0.17)	0.261	0.898	0.082

Data are presented as mean (95% CI of mean). HHb: deoxyhemoglobin; s: seconds; a.u.: arbitrary unit; StO_2_: tissue saturation *p < 0.05 for comparison between pre and post treatment, with Bonferroni correction; ^†^p < 0.05 for comparison between groups, via Bonferroni test.

**Table 3:** Comparison of mean changes of the NIRS variables between groups regarding the treadmill test, before and after the intervention.

Variables	Conventional Group		Modified Group		Effect group (p-value)	Effect situation (p-value)	Effect Interaction (p-value)
	Pre (n = 20)	Post (n = 16)	Pre (n = 20)	Post (n = 16)			
Delta HHb on treadmill (a.u.)	11.87 (7.96 to 15.78)	12.04 (6.75 to 17.34)	12.49 (8.67 to 16.31)	12.34 (7.18 to 17.5)	0.870	0.995	0.932
Delta StO_2_ on treadmill (%)	−19.09 (−22.77 to −15.41)	−18.00 (−22.30 to −13.69)	−21.07 (−24.65 to −17.49)	−21.29 (−25.47 to −17.11)	0.288	0.709	0.575
Time to reach lower StO_2_ on treadmill (s)	150.00 (109.72 to 190.28)	772.00^*,†^ (423.11 to 1120.89)	104.00 (64.74 to 143.26)	140.63 (−197.19 to 478.44)	0.008	0.010	0.020
Time of resistance after reaching lower StO_2_ (s)	133.16 (41.31 to 225.01)	1236.00* (487.78 to 1984.22)	147.50 (57.98 to 237.02)	1250.00* (525.54 to 1974.46)	0.956	<0.001	0.999
Recovery time on the treadmill (s)	720.68 (316.68 to 1124.68)	155.08* (72.54 to 237.62)	637.76 (210.66 to 1064.86)	223.77* (146.56 to 300.98)	0.962	0.002	0.610
Deoxygenation Rate (∆StO_2_/s)	0.19 (−2.60 to 2.99)	0.09^*,†^ (−1.73 to 1.91)	0.25 (−3.73 to 4.22)	0.20* (−1.29 to 1.7)	0.017	0.002	0.185
Re-oxygenation Rate (∆StO_2_/min)	−3.75 (−5.1 to −2.40)	−3.26 (−7.19 to 0.67)	−4.32 (−5.69 to −2.95)	−5.26 (−9.33 to −1.19)	0.375	0.870	0.601
Relative re-oxygenation rate (∆StO_2_/min)	−11.90 (−21.82 to −1.98)	−114.17* (−204.4 to −23.93)	−17.69 (−28.63 to −6.76)	−78.24* (−171.88 to − 5.4)	0.639	0.017	0.517
Walking economy meters/delta HHb (m/∆HHb)	16.61 (−35.35 to 68.56)	3.29 (−511.54 to 518.11)	−11.24 (−61.89 to 39.4)	−37.25 (−535.72 to 461.23)	0.847	0.912	0.972
Walking economy meters/delta StO_2_ (m/∆StO_2_)	−11.52 (−17.84 to −5.20)	−127.82* (−185.90 to −69.75)	−10.06 (−16.22 to −3.90)	−57.80* (−112.12 to −3.47)	0.078	<0.001	0.090

Data are presented as mean (95% CI). HHb: deoxyhemoglobin; a.u.: arbitrary unit; StO_2_: tissue saturation; s: seconds, ∆: delta/variation; *p < 0.05 for comparison between pre and post treatment, via Bonferroni test; ^†^p < 0.05 for comparison between groups, via Bonferroni test.

## Discussion

The present study is innovative as an assessment of the effects on muscle metabolism of overload training on the lower limbs compared to conventional walking, by means of adjustments of the NIRS. Our hypothesis was that patients with PAD who performed the modified aerobic with a load on the lower limbs would present greater gains in functional capacity, metabolism and muscular performance than subjects submitted to traditional aerobic walking training. Although differences in the muscular metabolic adaptive mechanisms were observed between the two groups, as verified by changes of the NIRS variables, both interventions resulted in improved walking capacity as evaluated by the treadmill test. A high adherence rate was observed, which was not different between the two intervention modalities. Despite the difference in body mass index (BMI) between the groups, both groups were in the overweight range. We believe that the possible clinical repercussions of being overweight were similar between the groups, so no adjustment was made in the statistical analysis for the variable BMI.

In both groups, there was a reduction of the relative recovery time on the treadmill, characterized by the ratio between the time required to return to the baseline StO_2_ and the total test time. Therefore, after the training the individuals took relatively less time to recover the baseline StO_2_ value as they walked longer. Our study corroborates the results of two other studies that verified the reduction of time required to recover StO_2_ after treadmill testing, following 12 weeks of training^32,33^. According to Beckit *et al*. (2012), the improvement in StO_2_ recovery after physical training reflects the combination of improvement in metabolic economy and muscle oxidative capacity. This suggests that exercise helps to reverse the metabolic myopathy acquired by individuals with PAD^33^. In addition, after the training, in both groups there was an increase in the time of resistance after reaching the lowest StO_2_. This finding indicates that after the intervention, the subjects were able to maintain walking exercise for a longer period of time, despite the level of muscle ischemia. The improvement of resistance after reaching the lowest StO_2_ reinforces the theory that training optimizes the transport and use of oxygen in patients with PAD^34^.

Both groups displayed improvement in the re-oxygenation rate relative to the total time for the treadmill test, characterized by the ratio between the StO_2_ variation during the test and the relative recovery time on the treadmill. Recovery after exercise reflects how much the oxygen supply exceeds the oxygen demand for muscle recovery^16^. As no change was observed in the variation of the StO_2_ after the intervention, the improvement in the rate of re-oxygenation is the result of a reduction in the time required for recovery of StO_2_ relative to the total test time. That is, for the same variation of StO_2_ during the test, the individual was able to recover faster, even having walked for longer after the intervention. The improvement in the re-oxygenation rate and the relative time of recovery suggest a probable optimization of both muscular oxidative capacity and vascular function^34^.

An improvement of the deoxygenation rate after the treatment was also observed in both groups. However, the conventional group presented a significantly higher result than the modified group. The deoxygenation rate is characterized by the ratio between the variation of StO_2_ during the test and the time to reach the lowest StO_2_ value. The improvement observed with regard to this variable implies a reduction in the velocity of deoxygenation induced by the exercise, indicating a probable improvement of the muscular oxidative capacity^33^. Although both groups showed an improvement in the deoxygenation rate, only the conventional group showed an increase in the time to reach the lowest StO_2_ on the treadmill after the intervention. This may have contributed to a higher magnitude of response in relation to the observed deoxygenation rate in the conventional group. Published studies have shown that both traditional walking and pole stride walking increased both the time required to achieve the lowest StO_2_ during the treadmill test and the walking capacity of individuals with PAD^35,36^. Another study also observed an increase in the time required to achieve the lowest StO_2_ during treadmill tests, in a group undergoing supervised walking training, and in a group that underwent unsupervised walking. This finding indicated that improvement in microvascular function as desaturation of oxygen-induced exercise was slower after training^32^. Tew *et al*.^37^ performed training with upper limb cycle ergometers in individuals with PAD, and also observed an increase in the time required to reach the lowest StO_2_ during exercise. Ischemia caused by the reduction of exercise-induced microvascular oxygenation is known to potentially cause the release of nitric oxide from the endothelium, therefore, improving vascular function^38,39^. The results found by these authors^37^, demonstrated an improvement in global vascular function, characterized by an improvement in the availability of oxygen to the lower limb during exercise that was detected by NIRS, even with training carried out using the upper limbs. Although in our study, the modified group did not show an improvement in the time to reach the lowest saturation on the treadmill, this group displayed an improvement in the distance. This result suggests that other adaptive mechanisms resulted in improved functional capacity in the modified group.

An improvement in walking economy relative to StO_2_, defined as the ratio between the number of meters traveled for each unit of StO_2_ fall, was observed in both groups. After the intervention the subjects were able to walk greater distances for each unit of StO_2_ fall. Although no statistically significant difference was detected between the groups after treatment, a clinically significant difference was observed in favor of the conventional group, with an increase in the walking economy by 11.09-fold versus a 5.75-fold increase in the modified group. The differences in the adaptations generated by the two types of interventions may justify the clinically significant difference in walking economy between the two groups.

The difference in the use of cilostazol between conventional and aerobic groups in this study may be considered a limitation. A recent meta-analysis found that cilostazol presents a negligible benefit in improving the maximum distance walked by individuals with PAD when compared to usual care. However, only exercise training was found to have a positive effect on the ability to walk^40^, Therefore, cilostazol, when compared with the usual care, has no positive effect on walking ability in individuals with PAD^40^. Although a decreased need for cilostazol was observed in the conventional group, the use of the drug did not influence adaptations to training, especially because there was no difference in walking ability among the groups.

The possible adverse effects expected from modified aerobic training were fatigue and muscular pain secondary to adding weights to the legs of people with PAD. However, none of these adverse effects were reported during the study demonstrating the viability of this type of training for PAD individuals. A pilot study was previously conducted to assess the viability and the progression of the aerobic training protocol with a load on the lower limbs in adults with PAD.

Conventional training resulted in greater optimization of the muscular oxidative capacity and vascular function in this study, since the CG presented an increase in the time to reach the lower StO_2._ Additionally, CG showed a greater improvement in the deoxygenation rate and a clinically significant difference in walking economy compared to MG. Although the two types of interventions resulted in different adaptive responses to training, as detected by NIRS, no difference in walking ability between the groups was observed. Despite different adaptive responses, the two groups showed significant functional improvement. The results of this study suggest that training has the potential to optimize microvascular perfusion and oxidative capacity, further increasing the availability of oxygen in the active fibers and resulting in functional improvement. The results of the present study are in agreement with previous studies performed on individuals with PAD, in which there was an increase in extraction capacity, muscle oxygen availability and functional capacity after training sessions^18,32,34–37^. These results, together with our data, suggest a relationship between muscle metabolic improvement and endothelial function, and clinical and functional improvement observed in individuals with PAD^34^.

## Conclusion

The present study demonstrated that traditional aerobic training was superior to modified walk training concomitant with overload in the lower limbs with respect to improvement of muscle metabolism. The conventional training resulted in a greater optimization of muscular oxidative capacity and vascular function in patients with PAD. However, the differences in the adaptive mechanisms did not result in differences in the walking ability among the groups after the intervention. The findings of this study broaden the understanding of the adjustments produced by two different types of exercise, and their utility in the optimization of rehabilitation programs for individuals with PAD, and for future studies.

## Supplementary information

Project summary

Consort check list
