# Supplementary material for: Effects of modified aerobic training on muscle metabolism in individuals with peripheral arterial disease: a randomized clinical trial
Source: Sci Rep. 2019 Nov 4;9:15966. doi: 10.1038/s41598-019-52428-7 (PMC6828812; doi:10.1038/s41598-019-52428-7)
Supplement: Supplementary file 1 — Project summary [file 41598_2019_52428_MOESM1_ESM.docx]

**Project summary**

**Background:** Peripheral arterial disease (PAD) is clinically characterized by intermittent claudication (IC). IC interferes directly in the functional performance of the individual. The gold standard for the treatment of patients with PAD is walking with a frequency of at least three times a week, minimum duration of 30 minutes and intensity close to the maximum IC level. Although muscle resistance training programs also represent a therapeutic option, the benefits of the training program using walking concurrently with the use of load are not described. **Objective:** The aim of this study was to present a protocol that compared the effects of conventional walking program and walking with weights on ankles, in adults with peripheral arterial disease (PAD) on functional capacity, muscular metabolism, muscular performance and heart rate variability. A randomized clinical trial was conducted with two groups: a conventional aerobic group and a modified aerobic group with weights on ankles. Both groups performed walking training 3 times a week for 12 weeks. Short Physical Performance Battery, Heel-Rise Test, treadmill test with constant speed and inclination, Incremental Shuttle Walk Test and Walking Impairment Questionnaire were used to assess the participants before and after the training period. Near infrared spectroscopy was used during exercise tests to verify exercise-induced muscle ischemia in real time. **Conclusion:** The results of the trial will be directly applicable to the development of new treatment strategies for adults with PAD.

**General information**

- Protocol title, protocol identifying number (if any), and date.

Effects of modified aerobic training on muscle metabolism in individuals with peripheral arterial disease: a randomized clinical trial

Trial registration: ISRCTN44928994.

Date of registration: 10/03/2011.

- Name and address of the sponsor/funder

- Fundação de Amparo à Pesquisa do Estado de Minas Gerais (FAPEMIG)

Av. José Cândido da Silveira, 1500 - Horto Florestal, Belo Horizonte – MG/Brasil, 31035-536

- Conselho Nacional de Desenvolvimento Científico e Tecnológico (CNPq)

SHIS QI 01, Conjunto B, Edifício Santos Dumont, Lago Sul – CEP 71.605-170 – Brasília – DF/Brasil

- Pró-Reitoria de Pesquisa da Universidade Federal de Minas Gerais.

Universidade Federal De Minas Gerais,Reitoria, 7° andar, Belo Horizonte - MG/Brasil, 31710-220

- Name and title of the investigator(s) who is (are) responsible for conducting the research, and the address and telephone number(s) of the research site(s), including responsibilities of each.

Danielle Aparecida Gomes Pereira, PhD

danielleufmg@gmail.com

Department of Physical Therapy, Universidade Federal de Minas Gerais. Avenida Presidente Antônio Carlos, 6627 - Pampulha, Belo Horizonte - MG, CEP 31270-901- Brazil

**Rationale & background information**

Peripheral arterial disease (PAD) is a chronic obstruction of the arteries that irrigate the extremities, and the main cause of PAD is peripheral atherosclerosis ^1^. The imbalance caused by obstruction between the supply and demand of tissue oxygen in individuals with PAD, which occurs during activities such as walking, is characterized by reduced tissue oxygenation ^2^. Intermittent claudication (IC) is characterized by pain, cramp, pressure, or weight sensation induced by ischemia during walking that is relieved with rest ^3, 4, 5^. Approximately 15 to 40% of individuals with PAD present with IC, which directly interferes with their functional performance ^6^.

Near infrared spectroscopy (NIRS) has been used to non-invasively assess exercise-induced muscle ischemia in real-time, using adjustments in the variables oxyhemoglobin (HbO_2_), deoxyhemoglobin (HHb), and tissue saturation (StO_2_) ^7^. Recent studies have shown that absolute StO_2_ values at rest are similar in healthy and in PAD subjects. However, during physical activity, individuals with PAD present a sudden drop of StO_2_ at the beginning of exercise, reach significantly lower values of StO_2_ and have a significantly longer recovery time compared with healthy individuals ^8^. Komiyama et al. demonstrated that the recovery time of StO_2_ in individuals with diabetes and PAD who were unable to complete a 5-minute treadmill test was significantly longer than the recovery time of individuals able to complete the same test, as verified by NIRS ^9^. Initial studies using NIRS in subjects with PAD suggest a probable relationship between functional performance and muscle metabolic responses ^8^.

The prescription of physical activity is widely used in primary prevention to control risk factors for cardiovascular diseases, including PAD ^4, 5, 10^. Physical exercise promotes an increase in the individual's functional capacity, increasing the walking time and distance before the onset of claudication and until maximal claudication ^1, 5, 10^. These effects are due to increased blood flow and nitric oxide synthesis, improved muscle oxidative capacity, attenuation of inflammation and ischemic muscle damage, and reduced energy expenditure for walking ^2, 10^.

The current basis for the treatment of patients with lower limb PAD through physical activity is to walk near the maximum level of claudication, with a frequency of at least three times a week for a minimum duration of thirty minutes and a minimum of three months ^10, 11, 12^. This type of treatment produces satisfactory functional results by increasing the time and distance walked by PAD patients ^10, 11^. Additionally, the pain threshold increases when patients participate in treatment with walking ^10, 11^. Muscle resistance training has also been a form of adjuvant intervention suggested for the treatment of patients with PAD ^13, 14^. Because of a sedentary lifestyle and decreased mobility, patients with PAD may have some degree of loss of muscle mass and the consequent ability to produce force, thus justifying the proposal of muscular resistance training ^5, 14^.

Mcguigan and colleagues ^13^ used a muscular strengthening program for patients with PAD and demonstrated increases in the claudication threshold, muscle capillarization, distribution of muscle fibers and amount of myosin, all in addition to an increase in distance walked by the patients. However, Hiatt et al. ^5^ demonstrated that a walking program produced superior results to strength training regarding the time of onset of intermittent claudication and total walking time. In their study, muscle strengthening did not result in further improvements in walking distance when performed after walking^5^. McDermott et al. ^14^ compared walking-type aerobic training to lower limb strengthening and observed that gains in walking performance were higher in the aerobic training group. However, the strengthening group obtained higher gains in the functional activity of climbing stairs ^14^. Ritti-Dias and colleagues ^15^ also compared whole body strength training to walking training and detected similar functional improvements. However, they observed that strength training produced less pain during the sessions, therefore increasing exercise tolerance ^15^. Parmenter et al. ^16^ observed that high-intensity resistance training for 24 weeks in elderly patients with PAD significantly improved walking distance in the six minute walk test. They found no improvements in the groups that underwent low intensity resistance training and unsupervised walking training ^16^.

It has been well established in the literature that walking treatment has a beneficial effect for individuals with PAD ^1, 4, 5, 10, 11^. Although muscle resistance training programs are a therapeutic option to increase walking distances for these patients, the benefits of a training program using walking concurrently with the use of load have not been described, although the association is feasible and promising. The evaluation of individuals with PAD with the aid of NIRS makes it possible to simultaneously analyze the effects of limiting or compensatory factors on perfusion, muscle metabolism and functional capacity. Given the alterations secondary to PAD, it is important to increase our knowledge of the chronic effects of the different types of training (aerobic and resisted muscle) on the muscular metabolic response.

**References**

^1^ Gardner AW, Katzel LI, Sorkin JD, et al. Exercise rehabilitation improves functional outcomes and peripheral circulation in patients with intermittent claudication: a randomized controlled trial. J Am Geriatr Soc**.** 2001; 49: 755-62.

^2^ Hamburg NM, Balady GJ. Exercise rehabilitation in peripheral artery disease: functional impact and mechanisms of benefits. Circulation**.** 2011; 123: 87-97.

^3^ Gardner AW, Montgomery PS, Killewich LA. Natural history of physical function in older men with intermittent claudication. J Vasc Surg. 2004; 40: 73-8.

^4^ Schainfeld RM. Management of peripheral arterial disease and intermittent claudication. J Am Board Fam Pract**.**  2001; 14: 443-50.

^5^ Hiatt WR, Wolfel EE, Meier RH, et al. Superiority of treadmill walking exercise versus strength training for patients with peripheral arterial disease. Implications for the mechanism of the training response. Circulation**.** 1994; 90: 1866-74.

^6^ Mcdermott MM, Guralnik JM, Greenland P, et al. Statin use and leg functioning in patients with and without lower-extremity peripheral arterial disease. Circulation. 2003; 107: 757-61.

^7^ Ferrari M, Muthalib M, Quaresima V. The use of near-infrared spectroscopy in understanding skeletal muscle physiology: recent developments. Philos Trans A Math Phys Eng Sci**.** 2011; 369: 4577-90.

^8^ Boezeman RP, Moll FL, Ünlü Ç, et al. Systematic review of clinical applications of monitoring muscle tissue oxygenation with near-infrared spectroscopy in vascular disease. Microvasc Res**.** 2016; 104: 11-22.

^9^ Komiyama T, Shigematsu H, Yasuhara H, et al. Near-infrared spectroscopy grades the severity of intermittent claudication in diabetics more accurately than ankle pressure measurement. Br J Surg. 2000; 87: 459-66.

^10^ Gardner AW, Poehlman ET. Exercise rehabilitation programs for the treatment of claudication pain. A meta-analysis. JAMA. 1995; 274: 975-80.

^11^ Watson L, Ellis B, Leng GC. Exercise for intermittent claudication. Cochrane Database Syst Rev. 2008; 4: CD000990.

^12^ Hirsch AT, Haskal ZJ, Hertzer NR, et al. ACC/AHA 2005 Practice Guidelines for the management of patients with peripheral arterial disease (lower extremity, renal, mesenteric, and abdominal aortic): a collaborative report from the American Association for Vascular Surgery/Society for Vascular Surgery, Society for Cardiovascular Angiography and Interventions, Society for Vascular Medicine and Biology, Society of Interventional Radiology, and the ACC/AHA Task Force on Practice Guidelines (Writing Committee to Develop Guidelines for the Management of Patients With Peripheral Arterial Disease): endorsed by the American Association of Cardiovascular and Pulmonary Rehabilitation; National Heart, Lung, and Blood Institute; Society for Vascular Nursing; TransAtlantic Inter-Society Consensus; and Vascular Disease Foundation. Circulation. 2006; 113: e463-654.

^13^ Mcguigan MR, Bronks R, Newton RU, et al. Resistance training in patients with peripheral arterial disease: effects on myosin isoforms, fiber type distribution, and capillary supply to skeletal muscle. J Gerontol A Biol Sci Med Sci. 2001; 56: B302-10.

^14^ Mcdermott MM, Ades P, Guralnik JM, et al. Treadmill exercise and resistance training in patients with peripheral arterial disease with and without intermittent claudication: a randomized controlled trial. JAMA**.** 2009; 301: 165-74.

^15^ Ritti-Dias RM, Wolosker N, de Moraes Forjaz CL, et al. Strength training increases walking tolerance in intermittent claudication patients: randomized trial. J Vasc Surg. 2010; 51: 89-95.

^16^ Parmenter BJ, Raymond J, Dinnen P, et al. High-intensity progressive resistance training improves flat-ground walking in older adults with symptomatic peripheral arterial disease. J Am Geriatr Soc**.** 2013; 61: 1964-70.

**Study goals and objectives**

The primary objective of this study was to evaluate the effects of a modified training program on muscle metabolism, using walking concomitant with the use of lower limb overload in individuals with peripheral arterial disease, and compare it to traditional aerobic walking with respect to limiting symptoms of ischemia. The secondary objective was to compare the functional capacity, muscular metabolism, muscular performance and heart rate variability response to the two types of training.

**Study Design**

This study was approved by the Research Ethics Committee of Universidade Federal de Minas Gerais on February 15, 2016 (CAAE registration 51274515.4.0000.5149) and was registered at http://www.isrctn.com (ISRCTN 44928994). All methods were performed in accordance with the relevant guidelines and regulations. All participants were duly informed about the study, including possible risks and benefits of the interventions, before signing the informed consent form. The present study is a randomized, single-blind clinical trial. It was not possible to blind the participants and the professionals responsible for the intervention due to the obvious difference between the two types of intervention. Therefore, only the evaluators were blinded and did not know the allocation of the individuals to the intervention groups. Participants were included in the study upon meeting the inclusion criteria. After selection and evaluation, the project coordinator referred the subjects to the professional responsible for the intervention, who, from a, block-generated sequential list, randomly allocated participants to one of two groups, the conventional aerobic group (CG) or the modified aerobic group concomitant with the use of lower limb (MG) overload. The project coordinator was responsible for generating the random allocation sequence, performed in blocks of four at [www.randomization.com](http://www.randomization.com).

**Methodology**

From February 2016 to March 2017 adults with PAD and intermittent claudication were enrolled in the study, from the Cardiology and Vascular Surgery Clinic, Clinical Hospital, Universidade Federal de Minas Gerais. Individuals with PAD were included in the study, regardless of sex, according to the following inclusion criteria: (1) presenting an ankle-brachial index (ABI) at rest of less than 0.9 and (2) no pain at rest. The exclusion criteria of the study were: (1) participation in a supervised exercise program in the last six months or (2) presence of diseases or complications that impeded training such as heart failure, unstable angina, arrhythmia, decompensated diabetes (capillary glycemia greater than 250 mg/dl), or signs of hemodynamic instability.

Measures

Two evaluations were performed, the first was prior to the intervention, and the second was at 12 weeks after supervised training. In the evaluation, in order to characterize the sample, clinical data were collected regarding the presence of diabetes mellitus, beta-blocker use, use of cilostazol, smoking, level of obstruction, presence of clinical signs of chronic venous insufficiency. The assessment was comprised evaluations of body mass index (Asimed®, Barcelona, Spain) and ankle brachial index (ABI) heart rate variability (HRV), answering a Walking Impairment Questionnaire (WIQ), and performing an Incremental Shuttle Walk Test (ISWT), a Short Physical Performance Battery (SPPB), a Heel-Rise Test (HRT), and a treadmill test with constant speed and inclination. The order of the four exercise tests were random. NIRS was used during exercise tests to evaluate the adjustments of the variables HbO_2_, HHb and StO_2_. NIRS (Artinis®, Portamon system, The Netherlands) was used to evaluate the adjustments of StO_2_ and HHb of the medial gastrocnemius muscle during the arterial occlusion maneuver, and during the treadmill test using Oxysoft software (Artinis®). After the ABI measurement, the NIRS sensors were positioned in the medial region of the gastrocnemius muscle at the level of the largest circumference, and fixed with plastic film and an elastic band. The data were initially obtained at a frequency of 10 Hz. With the individual positioned in the dorsal decubitus position after initial stabilization of the measurement, the baseline value of StO_2_ was recorded, and the arterial occlusion maneuver was initiated. This maneuver was performed with a cuff positioned between the medial and distal third of the individual's thigh. The cuff was inflated above 250 mmHg, but to a maximum of 280 mmHg, and maintained for a period of five minutes, until the measures stabilized. This procedure worked as a physiological calibration, creating a functional scale that enabled a better comparison between different individuals, as the variables HbO_2_ and HHb were provided by the software in arbitrary units. The NIRS device was maintained in the lower limb of the patient until recovery after the four exercise tests: ISWT, SPPB, HRT and treadmill test with constant speed and inclination.

The ISWT is a 12-stage, progressive, bi-directional walking test in which the velocity of the first stage is 30 meters per minute (m/min), and at each stage the velocity increases by 10 m/min. The test was conducted as previous described by Sing et al., 1992. At the end of the test, the individual was questioned regarding the subjective perception of exertion using the Borg scale. Blood pressure and heart rate were recorded, as well as ICT, ACT, ICD, ACD and resting time required for the symptoms to disappear.

The treadmill test protocol used a constant velocity of 3.2 km/h and a constant inclination of 10% to evaluate the functional capacity of individuals with PAD. Were recorded ICT, ACT, ICD, ACD and resting time required for the symptoms to disappear, as well as the ISWT. Individuals were instructed to walk as long as possible up to the maximum pain. One minute of warm-up was carried out on a treadmill, in which a progressive increase of speed and inclination will be made up to 3.2 km/h and 10%, until the start of the test. The test was performed until the ischemic limiting symptoms are reached. When the patient reached maximum pain, they performed a cool down with a velocity of 2.0 km/h and 0% inclination for one or two minutes, according to the tolerance of the individual, to avoid a sudden reduction in venous return with consequent reduction in cardiac output. We consider that the velocity of 3.2 km/h allowed us to assess both individuals who were more impaired and individuals with a lower functional deficit caused by PAD.

The HRT was performed with the individual standing barefoot, in orthostatism and with bipedal support. During the test, individuals performed the maximum number of plantar flexions they can bear up until the point of volunteer fatigue, as fast as possible. The evaluator registered the number of repetitions performed and the total time spent performing the test.

The SPPB is an instrument used for the assessment of functional capacity that combines data from a static balance test in orthostatism, gait velocity in a usual step and estimated muscle strength of the lower limbs, measured indirectly through the activity of sitting and standing from a chair. The protocol for the SPPB application was performed according to its Brazilian version, which includes tests of static balance in orthostatism, gait velocity and lower limb muscle strength.

The WIQ is used to obtain information about the perception of locomotion in individuals with PAD who present with intermittent claudication. The questionnaire assesses aspects related to the last month and consists of three domains: distance (distance that the individual can walk), speed (speed that the individual can walk) and stairs (number of stairs that the individual can climb). The WIQ presented validity and reproducibility after its translation into Portuguese in a Brazilian sample. The score ranges from zero to 100% in each domain, with 100% being the best functional score.

HRV is a simple, noninvasive and reliable technique that can be useful to evaluate the influence of the autonomic nervous system on the heart. For the evaluation of HRV during the tests, we used the Polar® heart rate monitor, model RS800CX. The intervals between two successive R waves (iRR) were recorded by means of a digital telemetry system consisting of a transmitter positioned on the participant’s chest and a heart rate monitor. After a 10-minute resting period with the participant performing spontaneous breathing in the dorsal decubitus, the HRV evaluation was initiated and the intervals between two successive R-waves (iRR) were recorded for an additional 10-minute resting period. The HRV was also evaluated in the exercise situation; that is, during the treadmill test and for 10 minutes immediately after stopping the test with the participant in a supine position.

Training protocols

The aerobic exercise training was performed three times per week, over a 12-week period by volunteers from both groups. The CG performed traditional training, while the MG performed training with progressive overload in the lower limbs. In both groups, the walking exercise was performed at an acceptable intensity until the limiting claudication symptom occurred, allowing periods of recovery. As soon as there was a resolution of the claudication symptoms, a new walk was begun. The time required for the resolution of symptoms between each walk was disregarded in recording the total activity time, which should have been 30 minutes. Warm-ups and recovery were performed at the beginning and end of each walk, respectively, by both groups. The following parameters in all sessions were recorded: time to symptom onset, time to the onset of limiting pain, rest time required for symptoms to disappear, and total distance walked by the volunteer in each session.

Conventional aerobic training

The aerobic training of the CG was started with walking on the floor for 30 minutes. From the moment that the individual ceased to report a limiting claudication symptom within 30 minutes, treadmill training on the treadmill without inclination, was started at the average velocity achieved during the last walking session on the floor. A progressive rise of 0.2 km/h in velocity was performed from the moment the individual ceased to report limiting symptoms within 30 minutes on the treadmill.

Modified aerobic training

The aerobic walking training performed by the MG included a progressive overload on the lower limbs through the addition of ankle weights. Training was started on the floor, at first without a load on lower limbs. The loads were added gradually, according to the load progression protocol. From the time the individual reached the minimum time of 15 minutes of walking on the floor without experiencing a limiting claudication symptom, ankle weights were added, progressively, adding 0.5 kilo in each lower limb up to 2 kilos and taking into account the time and the absence of a limiting symptom. From the moment that the individual reached the minimum time of 15 minutes with an overload of 2 kilos while walking on the ground without a limiting symptom, training was started on the treadmill. The treadmill training started with the average speed reached on the floor, but without overload on the lower limbs. Weights were added at the ankles to increase the overload, progressively, evolving from 0.5 kg in each lower limb up to 2 kg, in the same way as in the floor training. A progressive rise of 0.2 km/h in the speed of the treadmill was followed, if the individual did not reach limiting symptom within 15 minutes after the addition of the load of 2 kilos.

**Safety Considerations**

The study presented all the risks inherent in physical activity, such as falls, fatigue and muscular pain. However, the staff responsible for the study received training to deal with possible complications or adverse events.

#### Follow-Up

This study did not perform follow-up.

**Data Management and Statistical Analysis**

An independent researcher who was blinded to the group allocation performed database management and analyses. The Anderson-Darling test was used to evaluate the normal distribution of the continuous variables. The descriptive analysis of the data and the results are expressed as mean ± standard deviation, or as absolute and relative frequencies.

The comparisons of the categorical variables, to characterize the groups, were performed using Fisher's exact test (2x2 tables) or the Cramer's V coefficient (asymmetric tables). To evaluate the differences between the groups (conventional and modified), the situation (pre- and post-intervention treadmill tests), and the interaction between the groups and the situation, the Linear Mixed Model was used. The dependent variables "groups" and "situation" were entered into the model as fixed effects, and the "subjects" was entered as a random effect. For the choice of the best model, the values for the maximum restricted likelihood (-2 restricted log likelihood) were used, the diagonal covariance structure was used for the repeated measures, and the first order autoregressive structure was used for random effects. Compared to the intention-to-treat analysis, this method provides better estimates for lost data and addresses individual differences more adequately. An alpha value of 5% was set for statistical significance. The data were analyzed using the statistical software Statistical Package for the Social Sciences - (SPSS, Inc., USA, version 15.0).

Initially, the sample size was calculated considering the variable total distance on the treadmill test as the most important variable for the analysis. From previous studies of individuals with PAD with similar characteristics to the subjects allocated for this study28, the sample size calculated was 70 participants per group, setting a power of 80% and an alpha error of 5%. In a second moment, from a pilot study with 12 individuals in each group, the sample size was recalculated to represent the smallest size of effect for the estimation of the n sample size. To perform the calculation, an alpha error of 5% and a power of 0.80 were set, and the size of the effect of the ANOVA (f) was estimated by the equation: $\left( f=\sqrt{\frac{{SQ}_{A}}{{SQ}_{e}}} \right)$, where SQA= the sum of the squares of the respective source of variation and SQE = the sum of the squares of the errors. From the sample size calculation, 18 individuals were required per group. Considering the potential subject losses, 10% was added to the n calculated, totaling 20 individuals per group.

**Expected Outcomes of the Study**

The evaluation of individuals with PAD with the aid of NIRS makes it possible to simultaneously analyze the effects of limiting or compensatory factors on perfusion, muscle metabolism and functional capacity. Given the alterations secondary to PAD, it is important to increase our knowledge of the chronic effects of the different types of training (aerobic and resisted muscle) on the muscular metabolic response. The findings of this study will broaden the understanding of the adjustments produced by two different types of exercise, and their utility in the optimization of rehabilitation programs for individuals with PAD, and for future studies.

**Overall trial start date**

15/01/2016

**Overall trial end date**

01/02/2018

**Project Management**

D.P. conceived and designed research; G.S. analyzed data; D.M. was responsible for implementation of the study; D.P. and D.M. drafted manuscript; R.B. revised manuscript; D.P., D.M., G.S. and R.B. approved final version of manuscript.

**Ethics approval**

Ethics Committee of Research from UFMG, 15/12/2010, ref: registration ETIC 0559.0.203.000-10

**Publication and dissemination plan**

Planned publication in a high-impact peer reviewed journal.

The datasets generated during and/or analysed during the current study are/will be available upon request from Débora Pantuso Monteiro (deborapantuso@hotmail.com).

**Participant level data**

Available on request.
